# A Multiplex PCR for the Simultaneous Detection and Genotyping of the *Echinococcus granulosus* Complex

**DOI:** 10.1371/journal.pntd.0002017

**Published:** 2013-01-17

**Authors:** Ghalia Boubaker, Natalia Macchiaroli, Laura Prada, Marcela A. Cucher, Mara C. Rosenzvit, Iskender Ziadinov, Peter Deplazes, Urmas Saarma, Hamouda Babba, Bruno Gottstein, Markus Spiliotis

**Affiliations:** 1 Institute of Parasitology, University of Bern, Bern, Switzerland; 2 Graduate School for Cellular and Biomedical Sciences, University of Bern, Bern, Switzerland; 3 University of Monastir, Faculty of Pharmacy, Department of Clinical Biology B, Laboratory of Parasitology and Mycology, Monastir, Tunisia; 4 Instituto de Investigaciones en Microbiología y Parasitología Médica, Facultad de Medicina, Universidad de Buenos Aires, Paraguay 2155, Buenos Aires, Argentina; 5 Institute of Parasitology, University of Zurich, Zurich, Switzerland; 6 Department of Zoology, Institute of Ecology and Earth Sciences, University of Tartu, Tartu, Estonia; Queensland Institute of Medical Research, Australia

## Abstract

*Echinococcus granulosus* is characterized by high intra-specific variability (genotypes G1–G10) and according to the new molecular phylogeny of the genus *Echinococcus*, the *E. granulosus* complex has been divided into *E. granulosus sensu stricto* (G1–G3), *E. equinus* (G4), *E. ortleppi* (G5), and *E. canadensis* (G6–G10). The molecular characterization of *E. granulosus* isolates is fundamental to understand the spatio-temporal epidemiology of this complex in many endemic areas with the simultaneous occurrence of different *Echinococcus* species and genotypes. To simplify the genotyping of the *E. granulosus* complex we developed a single-tube multiplex PCR (mPCR) allowing three levels of discrimination: (i) *Echinococcus* genus, (ii) *E. granulosus* complex in common, and (iii) the specific genotype within the *E. granulosus* complex. The methodology was established with known DNA samples of the different strains/genotypes, confirmed on 42 already genotyped samples (Spain: 22 and Bulgaria: 20) and then successfully applied on 153 unknown samples (Tunisia: 114, Algeria: 26 and Argentina: 13). The sensitivity threshold of the mPCR was found to be 5 ng *Echinoccoccus* DNA in a mixture of up to 1 µg of foreign DNA and the specificity was 100% when template DNA from closely related members of the genus *Taenia* was used. Additionally to DNA samples, the mPCR can be carried out directly on boiled hydatid fluid or on alkaline-lysed frozen or fixed protoscoleces, thus avoiding classical DNA extractions. However, when using *Echinococcus* eggs obtained from fecal samples of infected dogs, the sensitivity of the mPCR was low (<40%). Thus, except for copro analysis, the mPCR described here has a high potential for a worldwide application in large-scale molecular epidemiological studies on the *Echinococcus genus*.

## Author Summary

The dog tapeworm *Echinococcus granulosus* (*E. granulosus*) is a cosmopolitan parasite. The adult worms reside in the small intestine of their definitive hosts (dogs). Infective eggs are shed with the feces into the environment and are orally ingested by intermediate hosts where they develop into the metacestode (larval) stage, causing cystic echinococcosis (CE) in humans and livestock. Ten intraspecific genotypes of *E. granulosus* (G1 to G10) have been reported from different intermediate host species. Based on the recently established molecular phylogeny, *E. granulosus* is now considered a complex consisting of four species: *E. granulosu*s *sensu stricto* (G1/G2/G3), *E. equinus* (G4), *E. ortleppi* (G5) and *E. canadensis* (G6–G10). Simple and highly discriminative molecular epidemiological approaches are needed to explore dynamics, life cycle patterns, and the pathogenicity of the members of this complex. We here introduce a one-step multiplex PCR (mPCR) protocol for the genotyping and discrimination of the different members of the *E. granulosus* complex, allowing three levels of discrimination: (i) *Echinococcus* genus, (ii) *E. granulosus* complex, and (iii) genetic variants within the *E. granulosus* complex. The relatively complicated task of *E. granulosus* complex speciation and genotyping is clearly simplified by mPCR, and this technique therefore represents a useful tool for routine practice.

## Introduction

Historically, four species have been recognized within the genus *Echinococcus*: *E. multilocularis*, *E. oligarthrus*, *E. vogeli* and *E. granulosus* [1]. *E. shiquicus* and *E. felidis* are two newly discovered additional species isolated from small Tibetan mammals and African lions, respectively [2], [3]. Extensive research on genetic variation, intermediate host affinities as well as morphological, biological and biochemical differences resulted in a more sophisticated classification of the dog tapeworm *E. granulosus* into ten genotypes/strains [4]–[6]: sheep strain (G1), Tasmanian sheep strain (G2), buffalo strain (G3), horse strain (G4), cattle strain (G5), camel strain (G6), pig strain (G7), cervid strain (G8), pig/human strain (G9) and Fenno-Scandian cervid strain (G10). The poorly characterized strain G9 is closely related to *E. canadensis* (G7) [7] and the existence of G9 as a separate genotype remains still controversial [8], [9].

More recently, new data obtained from phylogenetic analysis have shown an even more pronounced genetic divergence between these ten *E. granulosus* genotypes [5], [10]. Based on sequences of the complete mitochondrial genome [11] and several nuclear markers [8], [12], the phylogeny for *E. granulosus* was reconstructed. Data obtained from nuclear protein-coding genes resulting in two nuclear alternative phylogenies: (i) nuclear phylogeny [8] is supported by morphological data, whereas (ii) nuclear phylogeny [12] is in agreement with mitogenome phylogeny [13]. Thus, *E. granulosus* became considered as a complex consisting of four species: *E. granulosus sensu stricto* (G1/G2/G3), *E. equinus* (G4), *E. ortleppi* (G5) and *E. canadensis* (G6–G10). The phylogenetic relations within the latter group remain unresolved and are still under controversial discussion, since the *E. canadensis* cluster was proposed to be divided into the two species *E. canadensis* (G8/G10) and *E. intermedius* (G6/G7) [14], [15]. This proposal gained further support from nuclear phylogeny [8], but mitogenome phylogeny analyses contradicted this assumption by showing that *E. canadensis* (G6/G7/G10) form a subgroup and *E. canadensis* (G8) is a closely related sister taxon [16].

The adult worms of *E. granulosus* complex reside in the small intestine of their definitive hosts, principally wild or domestic canids. Infective eggs are shed with feces into the environment and are orally ingested by intermediate hosts where they develop into the metacestode (larval) stage, known as the aetiological agent of cystic echinococcosis (CE) in humans and predominantly ruminants, pigs and horses. Due to its success to undergo its life cycle in domesticated animals during both definitive and intermediate stages, *E. granulosus* constitutes an important worldwide public health problem with significant economic impact [17]–[19].

Human susceptibility to CE depends largely upon the infecting species or genotype of the *E. granulosus* complex. Worldwide molecular epidemiological studies revealed that *E. granulosus s.s.* (G1) is most commonly found in humans, but also a high prevalence of *E. canadensis* (G6) [20]–[25] and *E. canadensis* (G7) [26], [27] was reported. *E. ortleppi* (G5) has a very marginal impact on human health with only two reported cases [20], [28]. One major factor behind the worldwide spreading of many zoonoses can be the introduction of the parasite by host animals, as it happened in Australia, where *E. granulosus* was imported with domestic livestock about 200 years ago [29].

The worldwide distribution of CE reveals a geographic heterogeneity of *E. granulosus* species in many overlapping areas. Some examples are the co-existing genotypes *E. granulosus s.s.* (G1) and *E. canadensis* (G6) in North African countries [23], [30]–[32], *E. granulosus s.s.* (G1/G2), *E. ortleppi* (G5) and *E. canadensis* (G6/G7) in Argentina [20], [33], [34] or *E. granulosus s.s.* (G1), *E. canadensis* (G6) and *E. equinus* (G4) in Kyrgystan [35]. In these areas co-infections with more than one *E. granulosus* species/genotype might occur in the intermediate or definitive hosts. In addition, the not yet confirmed hypothesis of an eventual genetic exchange by sexual reproduction between *E. granulosus* species/genotypes is still discussed [36].

The knowledge about the distribution of the *E. granulosus* complex is important *e.g.* in the context of any control or eradication program. Thus, regular molecular epidemiological surveys provide key information on the spatio-temporal dynamics of parasite populations. Knowledge about the transmission and prevalence of *E. granulosus* in humans and animals, including dogs, is a basic step before and during control and/or surveillance strategies.

Different methods for genotyping genetic variants of the *E. granulosus* complex have been developed so far. Based on PCR amplified sequences of the mitochondrial *cytochrome c oxidase* subunit 1 *(cox1)* or the *NADH dehydrogenase* subunit 1 (*nad1*), genotyping can be performed in a relative time and/or cost intensive way by sequencing [37], RFLP (Restriction Fragment Length Polymorphism) [38], [39], fingerprinting [40] or SSCP (Single Strand Conformation Polymorphism) [41]. More recently, pure PCR based methods that simplify the genotyping have been designed. With a consecutive PCR approach a part of the *E. granulosus* complex (G1, G5, G6/G7) can be genotyped [42] and by applying four parallel PCRs the discrimination between *E. multilocularis*, *E. granulosus s.s.* (G1) and an *E. ortleppi* (G5)/*E. canadensis* (G6/G7) cluster is possible [43]. Parallel PCR approaches can be combined in a multiplex PCR setup and became rapidly and successfully applied worldwide in many aspects of DNA analyses, especially in the field of molecular diagnosis of infectious diseases such as bacterial [44], viral [45] and fungal [46] infections. For cestode infections, a 3-plex-PCR approach was already established to distinguish between *E. multilocularis*, *E. granulosus* complex and *Taenia* [47]. However, the potential of such an approach has not yet been evaluated for the specific detection and/or genotyping of different isolates within the *E. granulosus* complex.

Based on the identification of a number of discriminating polymorphism sites in nuclear and mitochondrial genes of the *Echinococcus* genus, we established a single-tube multiplex PCR (mPCR) approach that allows a rapid and simultaneous detection and discrimination among the following members of the *E. granulosus* complex: *E. granulosus s.s*. (G1/G2/G3), *E. equinus* (G4), *E. ortleppi* (G5), *E. canadensis* (G6/G7) and *E. canadensis* (G8/G10). We assessed the performance of the mPCR assay by re-identifying reference DNA panels (42 samples) and by genotyping 153 unknown DNAs from human and animal *Echinococcus* cyst samples isolated from infected intermediate hosts in Tunisia, Algeria and Argentina. Finally, we assessed the feasibility of applying mPCR for the detection and genotyping of *E. granulosus* complex in fecal egg samples, and directly in frozen or fixed parasite material (hydatid fluid or protoscoleces).

## Materials and Methods

### Strategy

Based on known mitochondrial or nuclear DNA sequences, polymorphisms between *Echinococcus* strains/genotypes were identified and used for strain/genotype specific primer design. Each primer pair was first applied on its respective genotype-specific DNA, and if one clear PCR product was amplified, it was applied on DNA samples of all other genotypes/strains in order to exclude non-specific amplicons. Finally, 11 primer-pairs resulting in genotype/strain/genus specific targets were used for the mPCR.

The mPCR was set up with normalized known template DNAs in a sequential approach by starting with one specific primer pair in the PCR mix, followed by the incorporation of other primer pairs. The PCR was run with every additional new primer pair on all genotype/strain specific DNA samples to confirm specificity. Simultaneously the molar amount of primers was adjusted in order to achieve comparable amplicon intensities.

To reduce variable parameters and to allow comparison between experiments the basic mPCR conditions using GoTaq DNA polymerase from Promega were defined as followed: 94°C for 3 min, 25 cycles of 94°C for 30 sec, 56°C for 30 sec, 72°C for 30 sec and a final extension step for 5 min at 72°C. With this setup the sensitivity range was determined by adding different amounts of template DNA into the mPCR mix. The specificity of the mPCR was tested by (i) adding more PCR cycles, (ii) using mixed DNA templates derived from different *Echinococcus* genotypes/strains, (iii) using template DNAs of closely related genus *Taenia* or (iv) by the addition of foreign DNA derived from bovine thymus or dog feces.

To exclude lab-specific conditions, 13 samples were genotyped by mPCR in two different laboratories. To assess potential problems with materials derived from different suppliers, the system was tested with DNA polymerases from different companies. The mPCR performance was further validated by genotyping 42 *E. granulosus* complex samples derived from known origin and genotype, and subsequently 153 unknown DNA samples were genotyped. Furthermore, the mPCR was assessed on DNA derived from *Echinococcus* eggs isolated from feces of infected dogs. Finally, approaches were developed to perform the mPCR directly on fresh protoscoleces, either frozen or fixed, or on hydatid fluid.

### Identification of DNA polymorphisms in gene sequences of different *Echinococcus* strains

Information on the complete mitochondrial genome sequences containing the genes *cytochrome oxidase subunit I* (*cox1*), *cytochrome oxidase subunit 2* (*cox2*), *ATP synthase subunit 6* (*atp6*) and *NADH dehydrogenase subunit I* (*nad1*) as well as mRNA sequences of the nuclear genes *RNA polymerase II* (*rpb2*), *DNA polymerase delta* (*pold*), *ezrin-radixin-moesin-like protein* (*elp*), *elongation factor 1 alpha* (*el1a*) and *calreticulin* (*cal*) were obtained from the databases of the National Center of Biotechnology Information (NCBI) for *E. granulosus s.s.* (G1/G2/G3), *E. equinus* (G4), *E. ortleppi* (G5), *E. canadensis* (G6/G7), *E. canadensis* (G8/G10), *E. multilocularis*, *E. vogeli* and *E. oligarthrus*. The respective sequences were retrieved via GenBank [http://www.ncbi.nlm.nih.gov/] and were aligned with BioEdit 7.0.9 to detect polymorphic sites. The accession numbers of the used *Echinococcus* sequences are listed at the end of the manuscript.

### Primer design

The primers were designed on the assumption that one specific 3′-base will be sufficient to result in genotype-specific amplification since Taq-polymerases lack a 3′–5′ proofreading activity. In consequence, primers were chosen such as to strain-specifically bind to the targets described above. If possible, primers were selected that contained more than one specific 3′-base, but five primers of the final set that were targeted to nuclear sequences matched this one base difference. Because genotyping based on single nucleotide polymorphisms (snips) is error-prone due to mutations [48], [49], we chose two genotype/strain-specific probes for all *E. granulosus* complex members. The exception was *E. canadensis* (G8/G10), where only one probe was selected due to its rare occurrence and close relationship to *E. canadensis* (G6/G7). Two additional probes were chosen: a common one for all *E. granulosus* complex members, and one for the overall detection of all known *Echinococcus* species: *E. granulosus* complex, *E. multilocularis*, *E. vogeli*, *E. oligarthrus* and *E. shiquicus*. Therefore, three levels of differentiation were obtained for each sample by determination of (i) the genus *Echinococcus*, (ii) the affiliation to the *E. granulosus* complex and (iii) the specific strain or genotype within the complex. For all primers, a Tm of approximately 55°C was selected, and for each primer-pair a PCR product of distinct size was anticipated, in order for the amplicons to be easily discriminated by 2% agarose gel-electrophoresis. Table 1 shows the complete list of the final 22 primers used in this study, including names, molar concentrations in the mPCR mix, the final product sizes, the specificities (genotypes), the primer sequences (including the polymorphic sites), the primer lengths, the target genes (gene marker), the accession numbers of the published DNA target sequences, and the corresponding positioning of the primer sequences within their targets.

**Table 1:** Characteristics of oligonucleotides used for *Echinococcus granulosus* complex multiplex PCR.

Primer name	Conc. in mPCR	Product size (bp)	Specificity	Sequence 5′–3′^a^	Primer lenght (bp)	Gene marker	Acc No (NCBI)	Primer position
*Echi* Rpb2 F	1 µM	1232	All *E.* species	TTGACCAAAGAAATCAGAC	19	*rpb2*	FN566850.1	55–74
*Echi* Rpb2 R	1 µM	1232	All *E.* species	CGCAAATACTCCATGG	16	*rpb2*	FN566850.1	1287–1271
*E.g* complex F	0.15 µM	110	*E. granulosus* complex	TGGTCGTCTTAATCATTTG	19	*cox2*	AF297617.1	10686–10705
*E.g* complex R	0.15 µM	110	*E. granulosus* complex	CCACAACAATAGGCATAA	19	*cox2*	AF297617.1	10796–10777
*E.g ss* cal F	2 µM	1001	*E. granulosus s.s.* (G1/G/G3)	CAATTTACGGTAAAGCA**T**	18	*cal*	U834931.1	151–169
*E.g ss* cal R	2 µM	1001	*E. granulosus s.s.* (G1/G/G3)	CCTCATCTCCACTCTC**T**	17	*cal*	U834931.1	1152–1135
*E.g ss* Ef1a F	1 µM	706	*E. granulosus s.s.* (G1/G/G3)	TCCTAACATGCCTTGGTA**T**	19	*ef1a*	FN568380.1	594–613
*E.g ss* Ef1a R	1 µM	706	*E. granulosus s.s.* (G1/G/G3)	GTTACAGCCTTGATCAC**G**	18	*ef1a*	FN568380.1	1300–1282
*E.eq* cal F	2 µM	426	*E. equinus* (G4)	GC**T**TATTTAGGATCCC**A**	17	*cal*	EU834936.1	566–583
*E.eq* cal R	2 µM	426	*E. equinus* (G4)	TCGTTTTTGCCAGT**G**	15	*cal*	EU834936.1	992–977
*E.eq* coxI F	0.2 µM	124	*E. equinus* (G4)	GTTGG**g**TT**g**GATGT**T**	15	*cox1*	M84664.1	143–158
*E.eq* coxI R	0.2 µM	124	*E. equinus* (G4)	CAAAAC**a**GGATCACT**CTT**	18	*cox1*	M84664.1	277–259
*E.ortp* ATP6 F	0.05 µM	1041	*E. ortleppi* (G5)	GTGTCGT**g**T**g**TTTA**g**T**G**A**G**	19	*atp-6*	AF235846.1	6057–6076
*E.ortp* ATP6 R	0.05 µM	1041	*E. ortleppi* (G5)	GCA**Ct**GA**T**A**ca**GG**t**GT**t**A**tT**	20	*atp-6*	AF235846.1	7098–7078
*E.ortp* CoxI F	0.2 µM	250	*E. ortleppi* (G5)	GGTT**t**TATGGGTTGTT**A**	17	*cox1*	AF235846.1	9978–9995
*E.ortp* CoxI R	0.2 µM	250	*E. ortleppi* (G5)	AC**A**CC**a**CCAAACGT**G**	15	*cox1*	AF235846.1	10228–10213
*E.cnd* G6/G7 pold F	1 µM	617	*E. canadensis* (G6/G7)	GGCCTTCATCTCCATAAT**A**	20	*pold*	FN568364.1	325–345
*E.cnd* G6/G7 pold R	1 µM	617	*E. canadensis* (G6/G7)	ATGAAGAGTTTGAAACTAAA**G**	21	*pold*	FN568364.1	942–921
*E.cnd* G6/G7 NDI F	0.3 µM	339	*E. canadensis* (G6/G7)	**c**TGCAGAGGTTTGC**C**	15	*nad1*	AB208063.1	7635–7650
*E.cnd* G6/G7 NDI R	0.3 µM	339	*E. canadensis* (G6/G7)	**c**ACAAC**aG**CA**t**AAAGC**G**	17	*nad1*	AB235847.1	7974–7957
*E.cnd* G8/G10 Elp F	1.5 µM	283	*E. canadensis* (G8/G10)	CCTAGTCTTCCCATGAT**A**	18	*elp1*	U834894.1	450–468
*E.cnd* G8/G10 Elp R	1.5 µM	283	*E. canadensis* (G8/G10)	ACAGAAGGCATATCC**A**	16	*elp1*	U834894.1	733–717

a ) Strict specific bases in each primer are written in bold. Tiny characters mark additional polymorphic sites (but not strict).

### mPCR conditions

**Figure 1:**
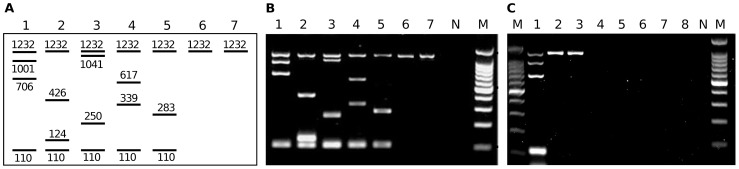
Genotype profile of the *E. granulosus* complex by mPCR. (**A**) Schematic representation of the genotype specific banding patterns amplified by mPCR: (lane 1) *E. granulosus s.s.* (G1/G2/G3), (lane 2) *E. equinus* (G4), (lane 3) *E. ortleppi* (G5), (lane 4) *E. canadensis* (G6/G7), (lane 5) *E. canadensis* (G8/G10), (lane 6) *E. multilocularis* and (lane 7) *E. vogeli*. The product sizes are specified in bp and the corresponding genes are shown in Table 1. (**B**) Result of a mPCR using 5 ng of purified template DNA of the known *Echinococcus* species described above (lanes 1–7) visualized on a 2% agarose gel. The target of 1232 bp is specific for the *Echinococcus* genus and is also amplified for *E. multilocularis* (lane 6) and *E. vogeli* (lane 7). The 110 bp band allows specific detection of *E. granulosus* complex members (lanes 1–5). All bands between 1232 bp and 110 bp specifically detected one *E. granulosus* complex species/genotype and showed no cross-reactivity with other members. (**C**) Specificity test of the mPCR for the genus *Echinococcus* and other closely related cestodes of the family; *E. granulosus* (G1/G2/G3) (lane 1), *E. multilocularis* (lane 2), *E. vogeli* (lane 3), *T. saginat*a (lane 4), *T. solium* (lane 5), *T. crassiceps* (lane 6), *T. taeniaformis* (lane 7) and *T. pisiformis* (lane 8). The expected banding pattern was observed for *E. granulosus* (G1/G2/G3) (lane 1), *E. multilocularis* (lane 2) and *E. vogeli* (lane 3) and no PCR products were detected for the *Taenia* samples. N: PCR-negative control (ddH_2_O). M: 100-bp DNA ladder (Promega).

The reaction mix for the final mPCR was composed of 100 µM dNTPs and 0.05 units µl^−1^ GoTaq DNA polymerase in 1× PCR Buffer (all Promega) and contained the 22 primers specific for 11 targets in the molarities shown in Table 1. For standard genotyping 5 ng template DNA were added into the PCR mix. Each reaction was performed in single tubes in a volume of 20 µl PCR mix. The cycling conditions were as follows: an initial denaturation step at 94°C for 3 min, 25 cycles (94°C–30 s, 56°C–30 s, 72°C–1 min) and a final extension step lasting 5 min at 72°C. 10 µl of the PCRs were separated by electrophoresis in a 2% agarose gel and visualized by ethidium bromide staining and subsequent UV excitation. The genotype specific amplicon profile is shown in Figure 1. The mPCR conditions were a result of pre-experiments described below, and these conditions were used throughout if not indicated otherwise.

### DNA samples, DNA extraction and DNA normalization

Ethical statement: For the parasite samples of animal origin, these were taken from animals in abattoirs being processed as part of the normal work of the abattoirs, in the frame of conventional meat inspection. For the parasite samples of human origin, these were obtained for and thus part of the normal diagnostic investigation to determine the etiology of the biopsied tissue for clinical purpose. Thus the present investigation was part of the conventional diagnostic procedure used in clinical practice. Samples were all anonymized for carrying out data evaluation.

**(A)** For establishment of the mPCR and all evaluations concerning the sensitivity and the specificity of the method, a test panel of *E. granulosus* complex chromosomal DNAs was used. Genomic DNA specimens used for the test panel were: *E. granulosus s.s*. (G1), *E. equinus* (G4), *E. canadensis* (G6), *E. canadensis* (G7), and *E. canadensis* (G8). These were obtained from institutional DNA-collections in Berne/Switzerland, Zürich/Switzerland and Tartu/Estonia. Genomic DNA extracted from *E. ortleppi* (G5) was kindly provided by Dr. Karen Haag (Departamento de Genétic, Instituto de Biociências, Universidade Federal do Rio Grande do Sul/Brazil) and protoscoleces from *E. canadensis* (G10) were kindly provided by Prof. Thomas Romig (Institute of Parasitology, University of Hohenheim/Germany). All samples had been genotyped conventionally by sequencing *cox1* and/or *nad1*. The genomic DNA of the *E. canadensis* (G10) protoscoleces was isolated using a standard phenol-chloroform protocol [50], using RNAse A (Sigma-Aldrich), Proteinase K (Sigma-Aldrich) and a subsequent isopropanol precipitation followed by multiple washes in 75% EtOH prior to drying and dissolving in ddH_2_O.

For most genotyped samples used in these parts of the study, the original extraction method for genomic DNA could not be retrospectively determined. A general problem in the usage of genomic DNA prepared by multiple methods (e.g. column based nucleic acid purification, phenol/chloroform extraction, presence or absence of RNAseA or proteinase K treatment) arises when quantifying the DNA concentration, e.g. by Nanodrop ND-1000 measurement. Therefore, an *E. granulosus s.s.* (G1) DNA amount (selected upon the most intense PCR amplification product when using the *Echinococcus* specific primers *Echi*-Rpb2 F and *Echi*-Rpb2 R, 1 µM, see Table 1), was defined as a reference measure point. The DNAs of all other species/genotypes were normalized to this sample by comparative PCR using the same primers. The PCRs were performed under the following conditions: 94°C for 3 min followed by 25 cycles of 94°C for 30 s, 56°C for 30 s and 72°C for 1 min and a final extension step of 5 min at 72°C.

**(B)** For the evaluation of specificity in the context of cross binding of the primers, DNA derived from *Echinococcus* species outside of the *E. granulosus* complex (*E. multilocularis* and *E. vogeli*) as well as DNA of the closely related *Taenia saginata*, *T. solium*, *T. crassiceps*, *T. taeniaformis* and *T. pisiformis* were obtained from the institutional DNA-collection at the University of Berne/Switzerland.

**(C)** For the evaluation of specificity in the context of contaminating DNA, bovine thymus DNA was obtained commercially from Serva, and dog feces DNA was isolated as described above by phenol/chloroform extraction from feces of a helminth-free dog that was obtained from the Small Animal Clinic of the Vetsuisse Faculty, University of Berne, Switzerland.

**(D)** For the assessment of the mPCR genotyping performance on DNA derived from metacestodes and/or protoscoleces, two panels of known (reference) and unknown *Echinococcus* metacestode DNAs were used. Known/genotyped materials were 20 reference DNA samples originating from Bulgaria [51] and 22 samples from Spain (unpublished) obtained from the institutional DNA-collection at the University of Berne/Switzerland. Unknown/non-genotyped materials were 13 DNA samples harvested from slaughterhouses in Buenos Aires/Argentina. Protoscoleces fixed in 95% (v/v) ethanol were obtained from 101 animal cysts harvested from slaughterhouses in Tunisia (75 samples) and Algeria (26 samples). Human isolates were collected after surgery from human patients in Tunisia (39 samples). Chromosomal DNA was prepared as described above. For more detailed information e.g. on host animal species, see Table 2. A part of these samples were used for the reliability and reproducibility tests. These 66 samples are marked with an asterisk in Table 2.

**Table 2:** Geographical origin, hosts and numbers of *E. granulosus* isolates and their corresponding species/strains based on multiplex-PCR results.

Region	Cyst origin	Number	Genotype
**North Africa**			
Tunisia	Ovine*	n = 75	*E. granulosus s.s.* (G1/G2/G3)
	Human*(3–15 yrs)	n = 39	*E. granulosus s.s.* (G1/G2/G3)
Algeria	Ovine	n = 22	*E. granulosus s.s.* (G1/G2/G3)
	Bovine	n = 4	*E. granulosus s.s.* (G1/G2/G3)
**Europe**			
Spain	Equid (horse)	n = 6	*E. equinus* (G4)
	Equid (donkey)	n = 1	*E. equinus* (G4)
	Ovine	n = 7	*E. granulosus s.s.* (G1/G2/G3)
	Bovine	n = 1	*E. granulosus s.s.* (G1/G2/G3)
	Human (adults)	n = 7	*E. granulosus s.s.* (G1/G2/G3)
Bulgaria	Bovine	n = 8	*E. granulosus s.s.* (G1/G2/G3)
	Ovine	n = 6	*E. granulosus s.s.* (G1/G2/G3)
	Porcine (pig)	n = 6	*E. granulosus s.s.* (G1/G2/G3)
**South America**			
Argentina	Porcine* (pig)	n = 7	*E. canadensis* (G6/G7)
	Ovine*	n = 6	*E. granulosus s.s.* (G1/G2/G3)
**Total**	animals	n = 149	
**Total**	humans	n = 46	

* *cox1*-sequenced samples: 14 ovine and 39 human Tunisian samples as well as the 6 ovine Argentinean samples were identified as *E. granulosus s.s.* (G1) and the 7 Argentinean porcine samples were identified as *E. canadensis* (G7).

**(E)** For the assessment of the mPCR genotyping performance of feces, eggs were isolated according to Mathis et *al.* [52] from 28 dog fecal samples (Sample collection Zürich/Switzerland: 20 samples from a study in Kyrgyzstan [35] and 8 samples from a study in Lithuania [53]). DNA extraction was performed as previously described [54], and the DNA was characterized by a multiplex PCR for the simultaneous detection of *E. granulosus* (G1–G10), *E. multilocularis* and *Taenia* spp. [47]. *Echinococcus* was identified in all samples; 18 out of 28 with *E. granulosus* (10 from Kyrgyzstan, mainly sheep strain G1) and 8 from Lithuania where only *E. canadensis* G7 occurs and 10 with *E. multilocularis* (10 from Kyrgyzstan). These preselected samples were used to assess the potential of the mPCR as a molecular diagnosis tool for canine infection with adult *Echinococcus*.

**(F)** To evaluate the mPCR directly on parasite material, none genotyped *Echinococcus* samples obtained from the institutional sample collection of Berne/Switzerland were used: (i) frozen hydatid fluid, (stored at −20°C) and (ii) solid *E. granulosus* complex germinal layers and protoscoleces, used natively (frozen) or fixed in either 95% (v/v) ethanol or 4% PBS-buffered formaldehyde solution.

### Pre-experiments and mPCR setup

The mPCR conditions described above were a result of 3 preliminary sets of experiments. Used samples are described above in sample section A.

- First, single primer pairs theoretically specific for one *E. granulosus* complex genotype/strain were applied at a concentration of 500 nM in a PCR mix containing 100 µM dNTPs and 0.05 units µl^−1^ GoTaq DNA polymerase in 1× PCR Buffer. As template 5 ng of the respective normalized DNAs were added (DNA normalization and template generation see below). Every PCR was performed in a final volume of 20 µl PCR mix in a 0.2 ml PCR tube. The cycling conditions were as follows: an initial denaturation step at 94°C for 3 min, 25 cycles (94°C–30 s, 56°C–30 s, 72°C–1 min) and a final extension step lasting 5 min at 72°C. Primer pairs resulting in single and clear genotype-specific amplicons were then screened for non-specific amplification products on the other genotypes/strains under identical conditions. Using the same approach, primer pairs detecting all *Echinococcus* species or only *E. granulosus* complex members were tested. In this step primer-pairs resulting in non-specific amplicons were discarded, or the specificity was increased by removing 5′-bases, leading to decreased annealing temperatures. The PCRs were performed as described above, and as depicted in Table 1, 22 primers for 11 specific amplicons were finally chosen for further studies.
- In a second set of preliminary experiments, the chosen single primer-pairs were tested for sensitivity and specificity range by addition of 10 pg, 100 pg, 1 ng or 5 ng of the different templates into the PCR mixes, and applying 25, 30 or 40 PCR cycles. As a result of this, 5 ng template DNA in a PCR setup with 25 cycles were chosen for establishment of the mPCR. The PCRs were performed as described in **(i)** and contained single genotype-specific primer-pairs and the respective template DNAs.
- In order to establish the mPCR, all 22 primers were combined in one mPCR mix but in different molar ratios to achieve a balanced and simultaneous amplification of all targets. Therefore, the amplicon yield was quantitatively normalized by adjusting the molar amount of the used primers. Starting with the *Echinococcus* genus specific primers *Echi* Rpb2 F and *Echi* Rpb2 R a molar primer concentration was determined, which resulted a clear but moderate amplicon yield when 5 ng of normalized template DNA of the different *Echinococcus* species were used in 25 cycle PCRs. In a sequential process, new primer pairs were added in different molarities into the mPCR mix, and the PCRs were performed in parallel with 5 ng normalized template DNA of the different *Echinococcus* species. The final primer concentrations of the mPCR mix resulting in similar amplicon intensities are shown in Table 1 and the resulting established standard mPCR conditions are described above.

### Assessment of sensitivity

To specify the amount of template DNA which can be used in the mPCR, the sensitivity of the method was determined by varying the template concentrations of normalized test panel *E. granulosus* complex DNAs in the standard mPCR mix containing all 22 primers. Therefore 0.1, 0.5, 1, 2.5, 5, 10, 25, 50, 100, 250, 500 ng and 1 µg normalized DNA from *E. granulosus s.s*. (G1), *E. equinus* (G4), *E. ortleppi* (G5), *E. canadensis* (G6), *E. canadensis* (G7), *E. canadensis* (G8) or *E. canadensis* (G10) were tested individually by mPCR employing the conditions described above (sample origin is described in sample section A). For the readout of this experiment low amounts of the different template DNAs had to result in clearly visible bands and high amounts of template should not yield additional or smeary products. With these preconditions/definitions a usable template range resulting in clear genotyping patterns was determined.

### Assessment of specificity

To test the influence of additional PCR cycles (more than 25), the mPCR was performed individually with 5 and 250 ng template DNA of the different *Echinococcus* strains (see sample section A). The mPCRs were run with 25, 30 and 35 amplification cycles and after gel electrophoresis the amplicons were screened for smeary or unspecific products to detect the cycle number range which resulted in clear genotyping patterns.

To determine the detection limit of a specific *E. granulosus* complex strain in a dual-strain DNA mixture, normalized test panel DNA from *E. granulosus s.s.* (G1) and *E. canadensis* (G6) were mixed and applied in the standard mPCR in total amounts of 5 ng (ratios; 80∶20, 60∶40 and 50∶50), 50 ng (ratios; 97.5∶2.5, 95∶5, 90∶10 and 80∶20) and 250 ng (ratios; 99.37∶0.63, 98.75∶1.25, 97.5∶2.5, 95∶5 and 90∶10). Samples are described in sample section A. For the readout, clearly visible amplicons of the *E. granulosus* complex DNA applied in lower ratios indicated a successful detection. Depending on the applied template amount, different ratios were detected. Additionally a DNA cocktail containing 5 ng of normalized test panel DNA from each member of the *E. granulosus* complex was used as template for the mPCR to verify that all 11 targets could be amplified simultaneously in one tube.

To exclude unspecific cross binding of the primers on the closely related *Taenia* genus, 10 ng template DNA derived from different *Taenia* species were applied in individually performed standard mPCRs. The samples employed for assessment of cross-binding are described above in sample section B.

To assess the mPCR specificity in the presence of host-derived contaminations in individually performed mPCRs, 5 ng of normalized *E. granulosus s.s.* (G1) test panel DNA (sample section A) were mixed with different amounts (1∶1 up to 1∶200) of two types of foreign DNA (sample section C). Clearly visible specific amplicons combined with a lack of unspecific PCR products indicated successful genotyping.

### Assessment of reliability and reproducibility

To confirm the reliability of the mPCR, a set of 66 samples (sample section D) were genotyped first according to the PCR-sequencing technique described by Bowles *et al.* 1992 [37], using the *cox1* primers JB3 (5′-TTTTTTGGGCATCCTGAGGTTTAT-3′) and JB4.5 (5′-TAAAGAAAGAACATAATGAAAATG-3′). The PCR products were purified with the High Pure PCR Product Purification kit (Roche Applied Science) according to the manufacturer's instructions and subsequently sequenced using an automated DNA sequencer (Applied Biosystems, ABI 3130× I Genetic Analyzer Sequencer). Sequence data were analyzed and compared with existing sequences derived from GenBank [http://www.ncbi.nlm.nih.gov/]. In a second step these 66 samples were used as templates in the standard mPCR setup using ∼20 ng DNA. Finally the results of both genotyping approaches were compared.

The reproducibility of the mPCR was assessed by performing the test in two different qualified laboratories and using the same mPCR protocol and test samples (see sample section D). Therefore, 13 samples from Argentina were genotyped in parallel by mPCR in the laboratories of Berne/Switzerland and Buenos Aires/Argentina. The mPCRs were performed with 20 ng template DNA as described above and the results were compared between the laboratories. Additionally, all 13 samples were genotyped by *cox1* sequencing (see above).

Since the mPCR was set up with GoTaq DNA polymerase from Promega and the DNA polymerases from different suppliers can influence the mPCR performance, a panel of DNA polymerases was tested in a second reproducibility test by replacing the GoTaq polymerase and GoTaq PCR buffer by other products in the standard mPCR setup. For the mPCR, 5 ng of normalized *E. granulosus s.s.* (G1) template DNA was used (Sample section A). DNA polymerase systems, which clearly yielded the 4 expected products, were designated as “useful” and the others yielding unspecific products, smears or missing amplicons were designated as “needing optimization”. The tested DNA polymerases and the performance results are listed in Table S1.

### Assessment of the mPCR genotyping performance

In total 195 *E. granulosus* complex DNA samples were tested. The DNA concentrations in all metacestode derived samples were measured and 1 µl (∼20 ng) of the DNA samples was used as template. The mPCR was performed with the standard settings described above. Information on the samples tested is given above in sample section D.

In order to investigate whether the mPCR is suitable as a molecular diagnostic tool to detect *Echinococcus* eggs in canine fecal samples, a panel of positively preselected DNA samples prepared from *Echinococcus* eggs was investigated. Since contaminating DNA can be present, 2 µl of the DNA samples (150–350 ng DNA) were used for mPCR, which was first performed under standard conditions as described above, and subsequently with 35 instead of 25 cycles and with up to 1 µg of template DNA per reaction. Information on the samples is given above in sample section E.

### Direct mPCR on frozen or fixed *E. granulosus* material

To simplify the genotyping procedure, we elaborated protocols that allow omitting DNA extraction procedures for mPCR amplification by using frozen or fixed *E. granulosus* materials (Sample section F). Many *Echinococcus* samples contain high amounts of calcium corpuscles that could interfere with the mPCR. These calcium corpuscles form a relatively solid pellet at the lowest bottom of the tube after centrifugation and by using the upper cellular part of the pellet a carry-over can be avoided. Frozen hydatid fluid (HF) (stored at −20°C) was thawed at room temperature and 1 ml was heated to 100°C for 30 min, centrifuged at 13,000 rpm for 10 min, and different volumes (0.25, 0.5, 1, 1.5, 2, 2.5, 3, 10 µl) of the resulting supernatant were used as templates for mPCR. Additionally, 1 and 2 µl none heated HF were applied in the mPCR.

Solid *E. granulosus* complex germinal layers (cut into small pieces) and protoscoleces were used either natively (frozen) or fixed, either in 95% (v/v) ethanol or 4% PBS-buffered formaldehyde solution. The material was prepared either by boiling or by alkaline lysis. In both cases, frozen material was used directly, and fixed material was pre-washed twice with PBS. For the preparation of the material by boiling, 10 µl solid sedimented *Echinococcus* material was resuspended in 90 µl H_2_O and incubated in a shaking heater (1,200 rpm, 100°C) for 30 min. Shaking is important in this step and if no shaking heater is available, the samples have to be vortexed from time to time, or must be intensively resuspended by pipetting. After centrifugation at 13,000 rpm for 10 min, different volumes (0.25, 0.5, 1, 1.5, 2, 2.5, 3, 10 µl) of the supernatant were used for the mPCR. For alkaline lysis, 10 µl solid *Echinococcus* material was incubated in 50 µl of 0.4 M NaOH and 2 µl of 1 M dithiothreitol (Sigma) and the mixture was heated for 15 min at 65°C in a shaking heater (1200 rpm). The suspension was neutralized by adding 50 µl of 0.4 M HCl and 1 µl 1.5 M Tris-HCl pH 8, and centrifuged for 10 min at 13,000 g. Shaking is important at this step (see above). For the mPCRs, 2 µl of different supernatant dilutions (1∶1, 1∶2, 1∶4, 1∶6, 1∶8, 1∶10 and 1∶25) were used in 20 µl setups. Furthermore, 1 and 2 µl undiluted supernatant were applied in the mPCR.

## Results

### Primer design and mPCR setup

The mitochondrial genome and different nuclear genes were aligned and analyzed for sequence differences appearing specifically within in the genes of the individual *E. granulosus* complex members: *E. granulosus s.s.* (G1/G2/G3), *E. ortleppi* (G4), *E. equinus* (G5), *E. canadensis* (G6/G7) and *E. canadensis* (G8/G10). Specific primer-pairs were designed and tested individually for sensitivity and specificity. In these preliminary experiments, primer concentrations were 0.5 µM, but template DNA amounts varied between 10 pg and 5 ng, and different numbers of amplification cycles (25, 30 or 40) were assessed. Primer pairs yielding specific and clear PCR products were combined to a set of 22 primers, which allowed the amplification of 11 different size-specific PCR products. This set of primers was used for the mPCR and the final concentrations of the primers in the mPCR mix were adjusted in order to achieve similar amplicon quantities. In this optimization step, 5 ng template and 25 amplification cycles were used, because by keeping the template DNA amount and amplification cycle numbers constant, the procedure for optimization of the final mPCR primer concentrations was simplified. In addition, keeping the numbers of cycles low reduced non-specific amplification and would speed up the procedure. The results of the single primer-pair tests that might be used for specific single primer-pair PCRs are depicted in Table S2 and all information about the chosen primers and their final concentrations used in the mPCR are shown in Table 1.

These pre-experiments resulted in a standard setup for the mPCR, which applies 22 primers at different concentrations. The mPCR was performed with GoTaq DNA polymerase in a final reaction volume of 20 µl and 25 amplification cycles. As template, 5 ng of normalized DNA of the different *E. granulosus* species were used. All reactions yielded a highly specific and clearly distinguishable banding pattern (Figure 1 A and B), allowing the discrimination among *E. granulosus s.s.* (G1), *E. equinus* (G4), *E. ortleppi* (G5), *E. canadensis* (G6/G7) and *E. canadensis* (G8/G10). The smallest band (110 bp) was designated to specifically indicate all members of the *E. granulosus* complex and was clearly present in all 5 species. The upper band (1232 bp) specifically identified the genus *Echinococcus* (Figure 1B) and detected the *E. granulosus* complex as well as *E. multilocularis* and *E. vogeli*.

### Sensitivity and specificity of the mPCR

**Figure 3:**
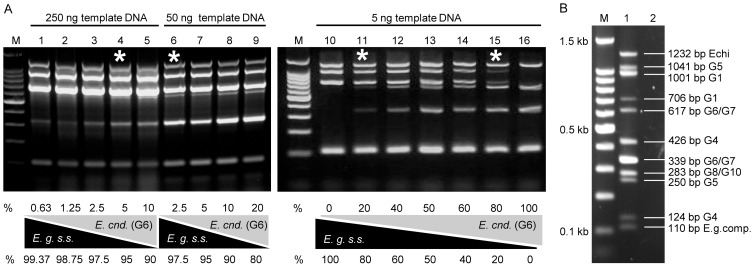
Detection limit of one species in a dual-species DNA mixture. **A**) To mimic a mixture of different *Echinococcus granulosus* complex members, as it can occur in egg-derived samples, DNA from *E. granulosus s.s.* (G1) and *E. canadensis* (G6) was mixed in different ratios and the mPCR was performed using 250 ng (lanes 1–5), 50 ng (lanes 6–9) or 5 ng (lanes 10–16) DNA template. The detection limit of one species in a dual-species DNA mixture was measured at 5% (lane 4, 250 ng template DNA), 2.5% (lane 6, 50 ng template DNA) and 20% (lanes 11 and 15, each 5 ng template DNA). **B**) To test if all 11 targets can be amplified in parallel, 5 ng template DNAs from *E. granulosus s.s.* (G1), *E. equinus* (G4), *E. ortleppi* (G5), *E. canadensis* (G6) and *E. canadensis* (G10) were mixed and used together in one single mPCR. All targets were successfully amplified and no missing or non-specific amplicon was detected (lane 1). Lane 2 shows the virtual banding pattern. Amplicon sizes and genotype specificities are marked on the left side. M: 100-bp DNA ladder (Promega).

The sensitivity of the mPCR was investigated by applying different concentrations of *E. granulosus* complex template DNA (0.1 ng–1 µg), and the results showed 5–250 ng template DNA are required for a successful detection of all members. When lower or higher amounts of DNA were employed, some PCR products were missing or non-specific amplification occurred. Out of the recommended amounts of template DNA, the detection limits depend largely on the species; *E. granulosus s.s.* (0.1 ng–1 µg), *E. equinus* (2.5 ng–250 ng), *E. ortleppi* (0.5 ng–250 ng), *E. canadensis* (G6/G7) (1 ng–500 ng) and *E. canadensis* (G8/G10) (5 ng–250 ng). Thus, in several experiments lower amounts of DNA (0.1–5 ng) were sufficient, but this occurred only when DNA of high quality was used (Figure 3A).

The specificity of the mPCR assay was investigated in four ways: (i) increasing the numbers of PCR cycles; (ii) employing mixed template DNA derived from different *Echinococcus* genotypes/strains; (iii) applying template DNAs of the closely related genus *Taenia*; (iv) addition of non-related DNA derived from bovine thymus or dog feces.

**Figure 2:**
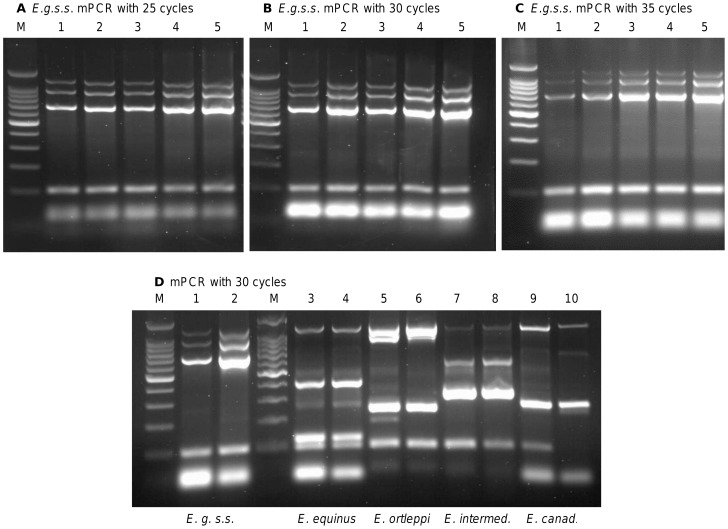
Specificity of the mPCR approach based on number of cycles. (**A–C**) Different quantities of *E. granulosus s.s.* (G1) DNA were used as templates in the mPCR: 5 ng (lane 1), 25 ng (lane 2), 50 ng (lane 3), 100 ng (lane 4) and 250 ng (lane 5). The mPCR was run with 25 cycles (**A**), 30 cycles (**B**) or 35 cycles (**C**) of amplification. For the *E. granulosus s.s.* (G1) template, the genotype was clearly detectable in all setups, but performing the mPCR with 30 or 35 cycles resulted in a visible background smear and some very light additional bands. A reduced setup was performed for the other genotypes (**D**). The mPCR was run with 5 ng (lanes 1, 3, 5, 7 and 9) or with 250 ng (lanes 2, 4, 6, 8 and 10) and 30 cycles of amplification. In contrast to *E. granulosus s.s.* (G1/G2/G3) (lanes 1 and 2) which showed only minor unspecific products, the mPCR amplified unspecific products for *E. equinus* (G4) (lanes 3 and 4), *E. ortleppi* (G5) (lanes 5 and 6), *E. canadensis* (G6/G7) (lanes 7 and 8) and *E. canadensis* (G8/G10) (lanes 9 and 10). Thus, additional numbers of PCR cycles result in unspecific PCR products hampering the readout. M: 100-bp DNA ladder (Promega).

Increasing the cycle numbers had an influence on the specificity of the mPCR. In the case where up to 250 ng normalized template DNA was applied, a specific banding pattern was achieved at 25 amplification cycles, but as shown in Figure 2, increased numbers of cycles still allowed genotyping based on the most prominent bands. However, in some genotypes, application of 30 cycles or more resulted in smeary or unspecific amplicons. Thus, for mPCR 25 amplification cycles are recommended.

To test the specificity of the mPCR with mixed template DNA derived from different *Echinococcus* species, two experiments were performed. First, a DNA cocktail containing 5 ng of normalized DNA from each member of the *E. granulosus* complex was used as template for the mPCR, and this resulted in a clear and simultaneous expression of all specific amplicons. Additionally, this experiment showed that all specific PCR products could be amplified in parallel, without interference or non-specific amplification (Figure 3B). Since *E. granulosus s.s.* (G1/G2/G3) and *E. canadensis* (G6/G7) have been reported to co-exist in several areas, these two species were selected to determine the detection limit of a specific genotype in a dual-genotype DNA mixture. Thus, DNA from *E. granulosus s.s.* (G1) and *E. canadensis* (G6) were mixed in different ratios and analyzed by mPCR. When 5 ng of the mixed DNA was used as template, one genotype could be detected when it was present in a concentration of 20% (Fig. 3A, lanes 11 and 15). By using 50 ng template DNA one genotype was detectable in a concentration of 2.5% (Fig. 3A, lane 6) and if 250 ng template DNA were used, the detection of one genotype was possible at a concentration of 5% (Fig. 3A, lane 4). Both experiments showed that two or more genotypes can be detected in parallel by mPCR.

To test the cross-reactivity with closely related *Taenia* species, mPCRs were performed with 10 ng template DNA of *T. saginata*, *T. solium*, *T. crassiceps*, *T. taeniaformis* and *T. pisiformis*. As shown in Figure 1C (lanes 4–8) no products indicative for non-specific primer binding were amplified.

**Figure 4:**
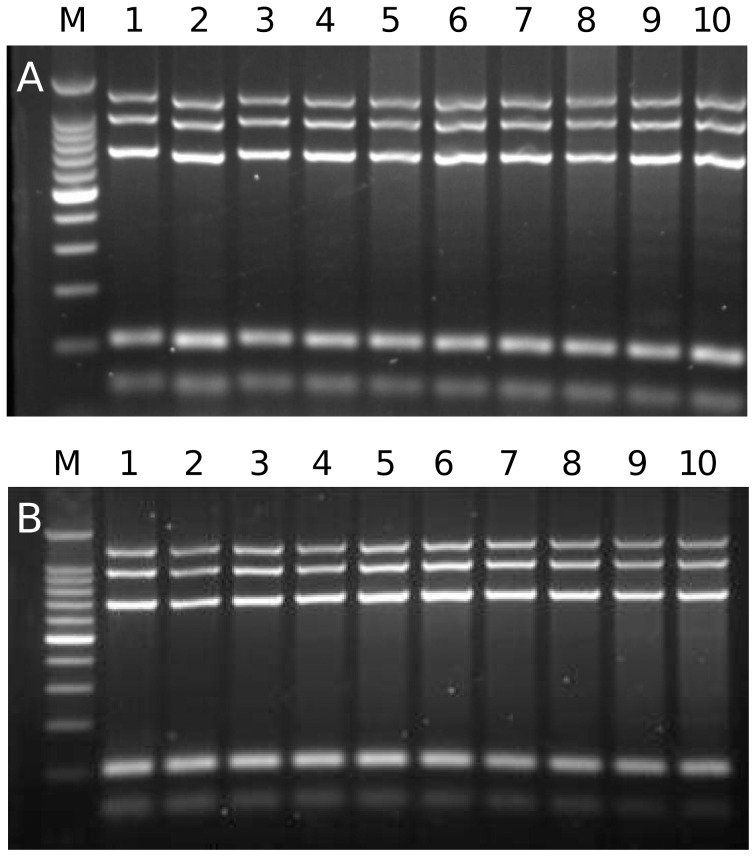
Specificity of the mPCR approach in contaminated samples. To mimic host derived contaminations of the template DNA, 5 ng of *E. granulosus s.s.* (G1) DNA was mixed in different ratios (1∶1 lane 1, 1∶2 lane 2, 1∶5 lane 3, 1∶10 lane 4, 1∶20 lane 5, 1∶30 lane 6, 1∶ 40 lane 7, 1∶ 50 lane 8, 1∶100 lane 9 and 1∶ 200 lane 10) with (**A**) DNA extracted from feces of a helminth-infection free dog and (**B**) calf thymus DNA. The background smear increased by applying more foreign DNA, but the genotype was still detectable, even when 1 µg total DNA was used as template (lane 9). M: 100-bp DNA ladder (Promega).

To mimic contaminations occurring during the isolation of DNA from metacestodes or *E. multilocularis* eggs, 5 ng normalized *E. granulosus s.s.* (G1) DNA and different amounts of DNA from bovine thymus or canine feces were mixed with at different rations (1∶1–1∶200). As shown in Figure 4, mPCR tolerated a 200-fold excess of foreign DNA (Figure 4).

### Reliability and reproducibility of the mPCR

To test the reliability of the mPCR, 66 unknown samples were genotyped by *cox1*-sequencing [37] and mPCR in parallel and both methods obtained identical results (Table 2, used samples marked with an asterisk).

The interlaboratory reproducibility of the mPCR was evaluated by genotyping 13 samples in parallel, namely in Berne/Switzerland and Buenos Aires/Argentina, respectively. Both laboratories employed GoTaq DNA polymerase, but otherwise worked independently from each other. Identical results were obtained; seven of the samples contained *E. canadensis* (G6/G7) and six contained *E. granulosus s.s.* (G1/G2/G3) isolates (data not shown).

In order to investigate whether the type of DNA polymerase used in mPCR could influence the results, a panel of DNA polymerases derived from different suppliers was tested. The GoTaq polymerase (Promega) originally used for the development of the mPCR yielded optimal results. However, similar results were obtained employing the 5× Multiplex PCR mix from New England Biolabs as well as AmpliTaq DNA Polymerase from Applied Biosystems. Other DNA polymerases failed to provide useful results, leading to non-specific amplicons, smeary products or missing amplification. A list showing the tested DNA polymerases is depicted in Table S1.

### Explorative study to assess the mPCR genotyping performance

The newly established mPCR was applied on previously characterized metacestode DNA, and on metacestode DNA samples of unknown origin. A total of 195 hydatid cysts, 149 isolated from animals and 46 obtained in humans, and all originating from different regions and/or continents, were genotyped by mPCR (for details on the samples, see Table 2). The mPCR amplified the corresponding genotype-specific banding patterns, and in no case unspecific amplicons or mixed genotypes were detected (data not shown). All 46 human CE cases and 135 of the 149 animal CE cases clustered within *E. granulosus s.s.* (G1/G2/G3). Furthermore, the mPCR detected 7 European *E. equinus* (G4) cases isolated from 6 horses and 1 donkey from Spain, and 7 pig-derived *E. canadensis* (G7) cases from South American samples (Table 2).

### mPCR on *Echinococcus* egg derived DNA samples

In this experiment 28 preselected *Echinococcus* egg DNA samples extracted from dog feces were used: 10 *E. granulosus s.s.* (G1), 8 *E. canadensis* (G7) and 10 *E. multilocularis* samples. Employing mPCR and 150–350 ng template DNA, only 5 out of 10 *E. granulosus s.s.* (G1) samples, 0 out of 8 *E. canadensis* (G7) samples, and 4 out of 10 *E. multilocularis* samples could be positively identified. Increasing the number of amplification cycles up to 35 and/or employing increased amounts of template DNA (up to 1 µg) did not result in any improvement (data not shown).

### Application of mPCR using fresh, frozen or fixed material

**Figure 5:**
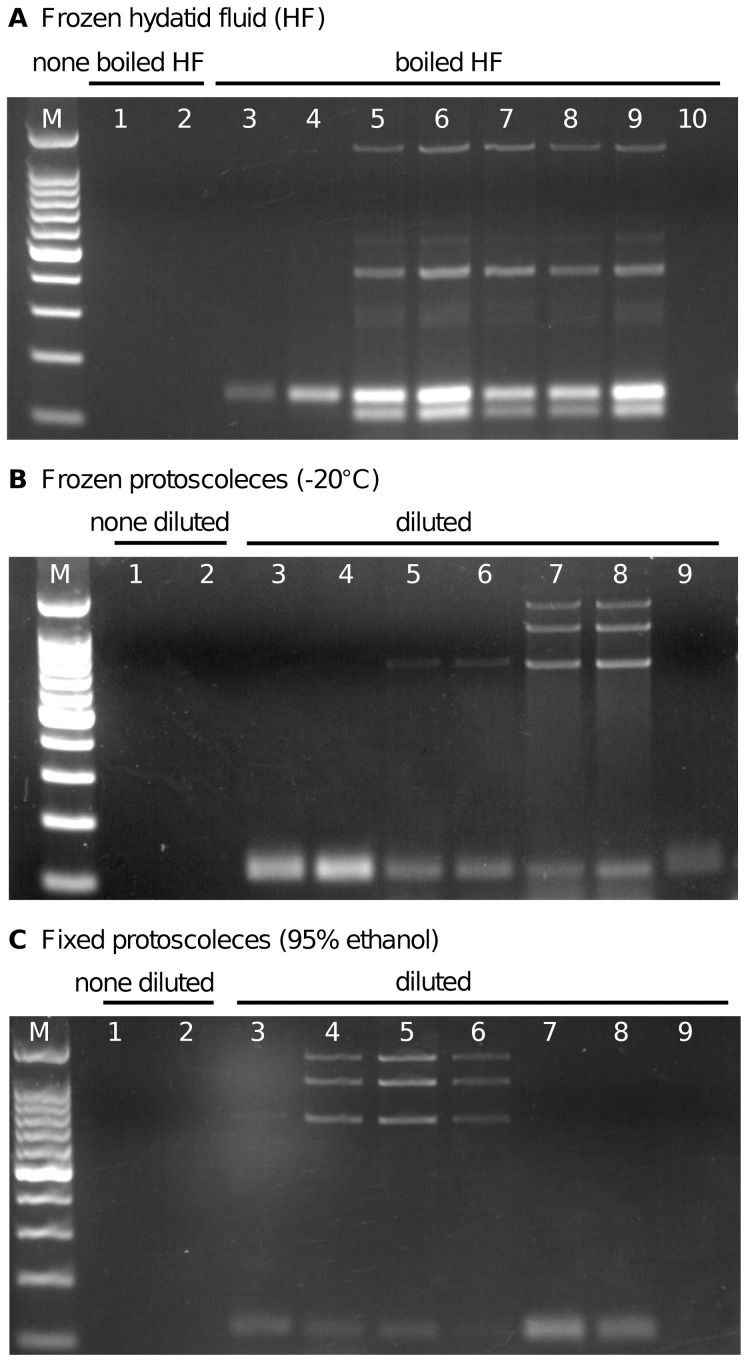
Direct mPCR on frozen and fixed *E. granulosus* complex material. (**A**) 1 µl (lane 1) or 2 µl (lane 2) of previously frozen hydatid fluid aspirated from an equid cyst was used directly in the mPCR without resulting in genotype specific PCR products. In parallel, 1 ml of the hydatid fluid was boiled for 30 min followed by a centrifugation step. Different volumes of the resulting supernatant were used in the mPCR (0.25 µl lane 3, 0.5 µl lane 4, 1 µl lane 5, 1.5 µl lane 6, 2 µl lane 7, 2.5 µl lane 8, 3 µl lane 9, 10 µl lane 10). Note that using 1–3 µl resulted in the detection of *E. equinus* (G4), although with some minor additional background amplicons. Frozen (**B**) and EtOH-fixed (**C**) *E. granulosus s.s.* (G1) protoscoleces were treated by alkaline lysis and the supernatant was used without (lanes 1 and 2) or with dilution (1∶1 lane 3, 1∶2 lane 4, 1∶4 lane 5, 1∶6 lane 6, 1∶8 lane 7, 1∶10 lane 8, 1∶25 lane 9) for mPCR. Undiluted supernatant (1 µl lane 1 and 2 µl lane 2) resulted in failed mPCR in both setups. If 2 µl of diluted supernatant was used for mPCR, genotyping was successfully performed for frozen protoscoleces on dilution ratios of 1∶8 to 1∶10 and for EtOH-fixed protoscoleces on ratios between 1∶2 to 1∶4. M: 100-bp DNA ladder (Promega).

In order to avoid time-consuming DNA extraction steps, the mPCR was performed directly on hydatid fluid (HF) and protoscoleces (Figure 5). The mPCR failed when these samples were used directly without any pre-treatment. However, heating HF followed by centrifugation and subsequent mPCR with 1–3 µl of the supernatant resulted in amplification of the entire *E. equinus* (G4) specific banding profile. Inclusion of lower or higher amounts of boiled HF supernatant, or inclusion of fresh, frozen or fixed *Echinococcus* tissue, did not result in mPCR amplification products (Figure 5A; data not shown). However, preparation of the material employing an alkaline lysis protocol resulted in effective genotyping with frozen and/or EtOH fixed samples, but not with protoscoleces fixed in 4% formaldehyde. When 2 µl of a 1∶8 or 1∶10 dilution of the alkaline lysed supernatant derived from frozen protoscoleces was used for mPCR the whole *E. granulosus s.s.* (G1) specific banding pattern was detected (Figure 5B). Application of 2 µl of a 1∶2 or a 1∶4 supernatant dilution of EtOH fixed protoscoleces resulted in the detection of a clearly amplified *E. granulosus s.s.* profile (Figure 5C). Conditions outside of these ranges yielded incomplete or lacking amplification of specific targets. It should be noticed at this point that calcium corpuscles interfere with the PCR. Best results were achieved when calcium corpuscles present at the bottom of the tube after centrifugation of the solid *Echinococcus* material were not included in the boiling or alkaline lysis steps.

## Discussion

The mPCR developed in this study represents an easy, rapid and inexpensive one-step detection method for the *E. granulosus* complex. This provides the unique opportunity to address directly speciation and genotyping within the framework of large-scale studies. However, as the *E. granulosus* complex at the genotypic level may considerably vary from region to region, we propose that routine control programs in a given area do not require the whole set of primers in the final mPCR mix as evaluated in our paper. Thus, a locally adapted primer combination may even render our approach strategy easier.

In the first step of the evaluation process, we defined a standard mPCR setup to minimize variable conditions. This setup enabled 100% specific amplification of targets for all *E. granulosus* complex members investigated. The setup was also successful when mixed genotypes or contaminating DNA from hosts (dog feces or cattle) were present. Additionally, the presence of closely related species such as other members of the genera *Echinococcus* or *Taenia* did not result in false positive amplification products. However, specificity diminished upon introduction of high amounts (>250 ng) of template DNA into the system or, conversely, when very low amounts (<5 ng) of template DNA were applied in combination with more than 25 rounds of PCR amplification. Highly reliable results were provided by using template DNA in the range of 5 to 250 ng. The method of DNA extraction could also have a substantial influence on the mPCR performance, since residual RNA, salt, ethanol or phenol could still be present, biasing DNA concentration measurement. Thus, for a standard genotyping experiment we finally recommend using 20–50 ng of template DNA per reaction, and for the simultaneous detection of different genotypes in one DNA sample up to 250 ng of template DNA should be applied. The same accounts for situations where a high contamination with foreign DNA is expected. In every case it should be taken into account that a minimal amount of specific *Echinococcus* DNA (approximately 5 ng) is necessary for the amplification. This is especially problematic when DNA extracted from eggs is used, as a single *Echinococcus* egg contains only approximately 8 pg of nuclear DNA [55], and therefore 600 *Echinococcus* eggs would be needed for reaching the minimal sensitivity threshold of one mPCR assay. As the worm burden of *Echinococcus* is highly dispersed in the dog population, the majority of animals are infected with low (<100) numbers of worms, which can result in relatively low egg numbers in the feces. In a study in Lithuania *E. canadensis* eggs were found in 9 of 240 dogs with egg numbers between 0.25 and 100 eggs per gram feces [52]. Therefore, for epidemiological investigations, the required amount of template DNA for the mPCR might be too high to reach the minimal amount of 5 ng *Echinococcus* DNA.

Nevertheless, the detection of canine echinococcosis is of essential interest since control programs are based mainly on the anthelmintic treatment of dogs, which interrupts the life cycle of the parasite. A highly sensitive PCR method to discriminate between the *E. granulosus* complex, *E. multilocularis* and other Taeniidae in fecal samples was established by Trachsel *et al.* [47]. This PCR is based on the amplification of mitochondrial genes employing a PCR setup with 40 amplification cycles, and thus low amounts of parasite DNA can be detected. However, genotyping of the *E. granulosus* complex is not possible with this approach. In comparison, the mPCR developed in this study could only detect 32% of those fecal samples that had tested positive by the PCR developed by Trachsel *et al.* [47]. Since those fecal samples had been pre-selected as *Echinococcus* PCR-positive from previous studies [35], [53], the real sensitivity of the mPCR might be even lower. Increasing template DNA concentration and increasing the numbers of PCR cycles did not result in improved sensitivity (data not shown). One possibility to apply the mPCR for the genotyping of canine derived samples would be the use of DNA extracted either from adult worms, isolated after necropsy or from purged dogs. With this approach 3 genotype groups (G1, G4 and G6/G7) were identified in dogs in Kyrgyzstan [35]. Another possibility would be the optimization of the mPCR protocol supporting the very low target amounts in a foreign DNA background, for example by using higher primer concentrations in a 30–40 amplification cycle setup.

So far, methods for genotyping *E. granulosus* complex members have been based on extracted DNA derived from protoscoleces (fertile cysts) or germinal layers (infertile cysts). In contrast to these methods, the mPCR protocol described here allows also genotyping without the need for DNA extraction steps, provided the material is frozen or fixed in ethanol. For solid materials such as protoscoleces, a direct testing, or testing upon pre-boiled treatment was not successful, but a pre-alkaline lysis step was sufficient for tissue dissolution and release of genomic DNA into the supernatant. Hydatid fluid of the metacestodes contains secretory parasite proteins and other metabolites derived from the germinal layer, and may also contain released and live germinal layer cells, and/or DNA derived from degraded cells. Thus, minimal amounts of boiled hydatid fluid can be used directly for mPCR-based genotyping. While this is possible with clear hydatid fluid, problems could occur in cases where the fluid is bacterially infected. In addition, application of excessive amounts of hydatid fluid or undiluted supernatants from boiled or alkaline-lysed *E. granulosus* might result in missing amplicons and therefore in imperfect genotyping. Nevertheless, compared to standard DNA extractions, both methods are fast, simple to perform and inexpensive. In our opinion, the most interesting finding was that the mPCR could be applied reliably with minimal amounts of boiled HF.

Another variable parameter of mPCR performance concerns the DNA polymerases. We optimized the protocol for GoTaq from Promega, but also Amplitaq (Applied Biosystems) or the Multiplex PCR 5× Mastermix (New England Biolabs) rendered good results, while the use of many other polymerases resulted in poor performance. In cases where other polymerases are used, the described protocols may have to be optimized.

In the explorative epidemiological application of our mPCR, a large amount of field samples obtained from different collaborating groups were investigated (Table 2). For all previously characterized isolates, the genotypes could be successfully confirmed by mPCR, including 20 samples from Bulgaria [51] and 22 samples from Spain (unpublished). All 176 samples derived from North African countries (Algeria and Tunisia; human and animal cases) were genotyped as *E. granulosus s.s.* by mPCR, and the 39 Tunisian human samples were additionally confirmed by *cox1* sequencing. In experiments carried out independently in two distinct laboratories, 13 Argentinean samples were genotyped by mPCR in Buenos Aires/Argentina and in Berne/Switzerland, and all results were comparable. These samples were additionally confirmed by *cox1* sequencing. Taken together 195 samples were genotyped, or the known genotype was confirmed by mPCR in this study. For all samples a clear genotype-specific banding pattern was observed, thus demonstrating the high accuracy of the *E. granulosus* complex mPCR. Compared to other genotyping methods (PCR-RFLP, sequencing or other approaches [56]–[59]) the mPCR resulted in similar findings, but results were obtained employing a rapid one-tube assay. Chromosomal DNA was used in this test-approach, but by applying hydatid fluid or cellular *Echinococcus* material as templates for the mPCR, the speed, price and hands-on-time for genotyping the *E. granulosus* complex can be further decreased. The relatively complicated task of *E. granulosus* complex speciation and genotyping is clearly simplified by mPCR, and therefore this method represents a useful tool for future routine practice.

In conclusion, the mPCR described herein represents a robust and reliable technique to characterize (i) any *E. granulosus* complex derived sample at the genus level, (ii) the membership within the *E. granulosus* complex and (iii) the species/genotype level, all in a single tube. Within the last two years, more than thirty studies addressed the question of genotyping of *E. granulosus* isolates around the world. This demonstrates the importance of the epidemiology of *Echinococcosis*, and the mPCR can contribute to a better understanding of the spatio-temporal circulation of this complex.

### Accession numbers of different *Echinococcus* sequences used for primer design

**A)** The primers *Echi* Rpb2 F and *Echi* Rpb2 R used for the detection of all *Echinococcus* species were designed using the *Echinococcus* gene *RNA polymerase II* (*rpb2*): *E. granulosus s.s.* (G1/G2/G3) - FN566850.1, *E. equinus* (G4) - FN566851.1, *E. ortl*eppi (G5) - FN566852.1, *E. canadensis* (G6) - FN566853.1, *E. canadensis* (G7) - FN566854.1, *E. canade*nsis (G8) - FN566855.1, *E. oligarthrus* - FN658827.1, *E. vogeli* - FN566847.1, *E. multilocularis* - FN566845.1. **B)** The complete mitochondrial genome sequence was used to design the *E. granulosus* complex specific primers *E.g.* complex F and *E.g.* complex R (gene marker: *cox2*), the *E. ortleppi* (G5) specific primers *E. ortp* ATP6 F and *E. ortp* ATP6 R (gene marker: *atp-6*) as well as *E. ortp* CoxI F and *E. ortp* CoxI R (gene marker: *cox1*) and the *E. canadensis* (G6/7) specific primers *E.cnd* G6/G7 NDI F and *E.cnd* G6/G7 NDI R (gene marker: *nad1*): *E. granulosus s.s.* (G1/G2/G3) - AF297617.1, *E. equinus* (G4) - AF346403.1, *E. ortleppi* (G5) - AF235846.1, *E. canadensis* (G6) - AB208063.1, *E. canadensis* (G7) - AB235847.1, *E. canadensis* (G8) - AB235848.1. **C)** The *ezrin-radixin-moesin-like protein* (*elp1*) was used to design the *E. canadensis* (G8/G10) specific primers *E.cnd* G8/G10 F and *E.cnd* G8/G10 R: *E. granulosus s.s.* (G1/G2/G3) - EU834886.1, *E. equinus* (G4) - EU834891.1, *E. ortleppi* (G5) - FN582298.1, *E. canadensis* (G6/G7) - EU834893.1, *E. canadensis* (G8) - EU834894.1, *E. canadensis* (G10) - EU834896.1. **D)** The *DNA polymerase delta* (*pold*) gene was used to design the *E. canadensis* (G6/7) specific primers *E.cnd* G6/G7 pold F and *E.cnd* G6/G7 pold R: *E. granulosus s.s.* (G1) - FN568361.1, *E. equinus* (G4) - FN568362.1, *E. ortleppi* (G5) - FN568363.1, *E. canadensis* (G6) - FN568364.1, *E. canadensis* (G7) - FN568365.1, *E. canadensis* (G8) - FN568366.1. **E)** The *calreticulin* (*cal*) gene was used to design the *E. granulosus s.s.* (G1/G2/G3) specific primers *E.g ss* cal F and *E.g ss* cal R as well as the *E. equinus* specific primers *E.eq* cal F and *E.eq* cal R: *E. granulosus s.s.* (G1) - EU834931.1, *E. equinus* (G4) - EU834936.1, *E. canadensis* (G6/G7) - EU834937.1, *E. canadensis* (G8) - EU834939.1, *E. canadensis* (G10) - EU834940.1. **F)** The *elongation factor 1 alpha* (*ef1a*) gene was used to design the *E. granulosus s.s.* (G1/G2/G3) specific primers *E.g ss* Ef1a F and *E.g ss* Ef1a R: *E. granulosus s.s.* (G1) - FN568380.1, *E. equinus* (G4) - FN568381.1, *E. ortleppi* (G5) - FN568382.1, *E. canadensis* (G6) - FN568384.1, *E. canadensis* (G7) - FN568383.1, *E. canadensis* (G8) - FN568385.1. **G)** The *cytochrome oxidase subunit I* (*cox1*) gene was used to design the *E. equinus* (G4) specific primers *E.eq* cox1 F and *E.eq* cox1 R: *E. granulosus s.s.* (G1/G2/G3) - M84661.1, *E. equinus* (G4) - M84664.1, *E. ortleppi* (G5) - M84665.1, *E. canadensis* (G6) - M84666.1, *E. canadensis* (G8) - DQ144021.1, *E. canadensis* (G10) - DQ144022.1.

## Supporting Information

Table S1 DNA polymerases tested in the *Echinococcus granulosus* complex multiplex PCR.
(DOCX)

Table S2 Sensitivities and specificities of single primer-pairs (each 0.5 µM) used in genotype specific PCRs.
(DOCX)
