# Supplementary material for: A Multiplex PCR for the Simultaneous Detection and Genotyping of the Echinococcus granulosus Complex
Source: PLoS Negl Trop Dis. 2013 Jan 17;7(1):e2017. doi: 10.1371/journal.pntd.0002017 (PMC3547860; doi:10.1371/journal.pntd.0002017)
Supplement: Table S1 — DNA polymerases tested in the Echinococcus granulosus complex multiplex PCR. (DOCX) [file pntd.0002017.s001.docx]

**Table S1:** DNA polymerases tested in the *Echinococcus granulosus* complex multiplex PCR

| **Working/optimization** | **Name** | **Supplier** | **Cat. No.** |
| --- | --- | --- | --- |
| DNA polymerases used in this study | AmpliTaq DNA Polymerase | [Applied Biosystems](http://www.google.ch/url?q=http://www6.appliedbiosystems.com/support/techtools/pcropt/&sa=U&ei=_Q20Tr_DEcja4QSppcHVAw&ved=0CBYQFjAB&usg=AFQjCNFYl_y82ILXYWp3doOKDgxbUkIlTA) | N8080160 |
|  | GoTaq DNA Polymerase | Promega | [M3001](http://www.promega.com/products/pcr/routine-pcr/gotaq-amplification-family/) |
|  | Multiplex PCR 5 × Master Mix | New England BioLabs | M0284S |
|  |  |  |  |
| DNA polymerases needing optimization | Expand High Fidelity PCR System | [Roche Applied Science](http://www.google.ch/url?q=http://www.lablife.org/p%3Fa%3Dproducts_show%26id%3D323681&sa=U&ei=mCC0TublAaqg4gSJtoTeAw&ved=0CBwQFjAE&usg=AFQjCNGdmqzndyBQVIX-us3nom6u6sKm1g) | 11732650001 |
|  | FIREPol DNA Polymerase | [Solis BioDyne](http://www.google.ch/url?q=http://www.sbd.ee/index.php%3Flan%3DEN%26sid%3D115%26tid%3D81%26active%3D62%26active1%3D133&sa=U&ei=hBG0Tqv8B-r54QSRnoXaAw&ved=0CBMQFjAB&usg=AFQjCNEgtNWmag5r9_fGFPihDp6fOj1Ycw) | 01-01-00500 |
|  | [GoTaq Flexi DNA Polymerase](http://www.google.ch/url?q=http://www.promega.com/resources/protocols/product-information-sheets/g/gotaq-flexi-dna-polymerase-m829-protocol/&sa=U&ei=CBK0Tt2qHI774QSR9fzsAw&ved=0CBEQFjAA&usg=AFQjCNEJPidmANQYAuUiNIrnoGq1M3ys3A) | Promega | M8291 |
|  | GoTaq Hot Start Polymerase | Promega | [M5001](http://www.promega.com/products/pcr/routine-pcr/gotaq-amplification-family/) |
|  | HotStarTaq Master Mix Kit | Quiagen | 203443 |
|  | One Taq 2X Master Mix | New England BioLabs | M0479G |
|  | One Taq DNA Polymerase | New England BioLabs | M0480G |
|  | One Taq HOT Start DNA Polymerase | New England BioLabs | M0481G |
|  | Pfu DNA Polymerase | Promega | [M7741](http://www.promega.com/products/pcr/routine-pcr/pfu-dna-polymerase/) |
|  | Taq DNA Polymerase (Taq Buffer) | New England BioLabs | M0273G |
|  | Taq DNA Polymerase (ThermoPol Buffer) | New England BioLabs | M0267S |
|  | Taq DNA Polymerase Brasil | Invitrogen | 11615-010 |
