# Supplementary material for: A Multiplex PCR for the Simultaneous Detection and Genotyping of the Echinococcus granulosus Complex
Source: PLoS Negl Trop Dis. 2013 Jan 17;7(1):e2017. doi: 10.1371/journal.pntd.0002017 (PMC3547860; doi:10.1371/journal.pntd.0002017)
Supplement: Table S2 — Sensitivities and specificities of single primer-pairs (each 0.5 µM) used in genotype specific PCRs. (DOCX) [file pntd.0002017.s002.docx]

**Table S2:** Sensitivities and specificities of single primer-pairs (each 0.5 µM) used in genotype specific PCRs.

| **template** | 10 pg | 10 pg | 10 pg | 100 pg | 100 pg | 100 pg | 1 ng | 1 ng | 1 ng | 5 ng | 5 ng | 5 ng |
| --- | --- | --- | --- | --- | --- | --- | --- | --- | --- | --- | --- | --- |
| **PCR cycles** | 25x | 30x | 40x | 25x | 30x | 40x | 25x | 30x | 40x | 25x | 30x | 40x |
| **Primers** |  |  |  |  |  |  |  |  |  |  |  |  |
| Echi Rpb2 F/R | - | - | ++ | + | + | ++a | + | ++ | +++ | + | ++s | +++s |
| E.g ss cal F/R | - | - | +a | + | + | ++a | + | ++ | +++ | + | ++s | +++a |
| E.g ss Ef1a F/R | - | - | - | +a | +a | +a | +a | ++a | ++ | + | ++s | +++a |
| E.eq cal F/R | - | - | - | +a | +a | +a | +a | ++a | ++a | + | ++ | ++a |
| E.eq coxI F/R | + | + | ++ | ++ | ++ | ++ | ++ | ++s | ++s | ++ | ++ | ++a |
| E.ortp ATP6 F/R | + | ++ | +++s | +++ | +++ | +++ | ++ | +++s | +++s | ++ | +++ | +++s |
| E.ortp CoxI F/R | + | ++ | +++ | ++ | ++ | +++ | ++ | ++ | +++s | + | ++ | +++ |
| E.cnd G6/G7pold F/R | - | + | + | - | + | ++ | + | + | +++a | + | + | ++a |
| E.cnd G6/G7 NDI F/R | - | + | ++a | ++ | ++ | +++ | + | +++ | +++s | + | ++ | +++ |
| E.cnd G8/G10 Elp F/R | - | + | ++a | + | + | +++a | + | ++ | +++ | + | ++ | +++s |
| E.g complex F/R | + | + | ++a | +a | +a | ++a | +a | n | n | + | n | n |

+ = low to moderate amplification yield; ++ = good amplification yield; +++ = high amplification yield; s = useful for genotyping but smears; a = additional bands but allowing genotyping; n = no genotyping due to non-specific amplification; - = no amplification products
